# The differential impact of economic recessions on health systems in middle-income settings: a comparative case study of unequal states in Brazil

**DOI:** 10.1136/bmjgh-2019-002122

**Published:** 2020-02-28

**Authors:** Lucas Salvador Andrietta, Maria Luiza Levi, Mário C Scheffer, Maria Teresa Seabra Soares de Britto e Alves, Bruno Luciano Carneiro Alves de Oliveira, Giuliano Russo

**Affiliations:** 1 Departamento de Medicina Preventiva, Faculdade de Medicina, Universidade de Sao Paulo, Sao Paulo, São Paulo, Brazil; 2 Centro de Engenharia, Modelagem e Ciências Sociais Aplicadas, Universidade Federal do ABC, Santo Andre, São Paulo, Brazil; 3 Departamento de Saúde Pública, Universidade Federal do Maranhão, Sao Luis, Maranhão, Brazil; 4 Centre for Primary Care and Public Health, Queen Mary University of London, London, United Kingdom

**Keywords:** economic crisis and health, recession and human resources for health, health systems in LMICs, Brazil, health financing

## Abstract

**Introduction:**

Although economic crises are common in low/middle-income countries (LMICs), the evidence of their impact on health systems is still scant. We conducted a comparative case study of Maranhão and São Paulo, two unevenly developed states in Brazil, to explore the health financing and system performance changes brought in by its 2014–2015 economic recession.

**Methods:**

Drawing from economic and health system research literature, we designed a conceptual framework exploring the links between macroeconomic factors, labour markets, demand and supply of health services and system performance. We used data from the National Health Accounts and National Household Sample Survey to examine changes in Brazil’s health spending over the 2010–2018 period. Data from the National Agency of Supplementary Health database and the public health budget information system were employed to compare and contrast health financing and system performance of São Paulo and Maranhão.

**Results:**

Our analysis shows that Brazil’s macroeconomic conditions deteriorated across the board after 2015–2016, with São Paulo’s economy experiencing a wider setback than Maranhão’s. We showed how public health expenditures flattened, while private health insurance expenditures increased due to the recession. Public financing patterns differed across the two states, as health funding in Maranhão continued to grow after the crisis years, as it was propped up by transfers to local governments. While public sector staff and beds per capita in Maranhão were not affected by the crisis, a decrease in public physicians was observed in São Paulo.

**Conclusion:**

Our case study suggests that in a complex heterogeneous system, economic recessions reverberate unequally across its parts, as the effects are mediated by private spending, structure of the market and adjustments in public financing. Policies aimed at mitigating the effects of recessions in LMICs will need to take such differences into account.

**Key questions**

**What is already known?**

- The literature suggests that population health and health systems are generally affected during economic recessions, but not much is known about large low/middle-income countries (LMICs), where systems are pluralistic and economic conditions very uneven.

**What are the new findings?**

- Our data show the crisis has had a diverse impact in São Paulo and Maranhão; in the former the macroeconomic slowdown has been more intense, with greater job losses, rising poverty levels, decrease of public spending as well as private insurance coverage, but increase of private health spending.
- In Maranhão the crisis was felt less intensely; the deterioration of the macroeconomic conditions was less noticeable, public health spending maintained its pre-crisis level, sustained by the state’s own resources and decentralised funds, and private insurance coverage kept steady.

**What do the new findings imply?**

- If confirmed, this ‘differential impact’ of recessions in LMICs would imply that the more dynamic and sophisticated parts of health systems find themselves more exposed during an economic downturn than poorer systems that rely predominantly on less volatile public funds.
- In such a scenario, public health spending would create mechanisms of social protection during an economic downturn, therefore counteracting the expected disproportionate impact of recessions on services for the poor.

## Introduction

Economic recessions are a recurrent phenomenon in high and low/middle-income countries (LMICs). Following the 2008 financial crisis in the USA, world economies experienced a period of economic instability and deteriorating health outcomes, which spread first to Europe, the so-called ‘Great Recession’^1^; Africa^2^; and more recently to South America.^3^ There is enough evidence to show that economic recessions do have an impact on the health of the population^4 5^ and on health systems, although evidence for the former is less straightforward.^6 7^

Fiscal austerity^8^ is a typical policy response of governments in the face of debt and economic crises—a set of restrictive measures with the stated objectives of reducing public expenditures, balancing budgets and promoting efficiencies in the public sector—although evidence shows that these objectives are often unfulfilled. Health sectors have been affected during economic recessions from the combination of increased demand for specific services, restrictive nationwide economic policies and health sector-specific measures^9^; however, such effects reverberate differently through the health system, as they are influenced by local determinants of health and supply-side heterogeneity.^10^

Large middle-income countries across the world are often far from being economically homogeneous entities and present very diverse combinations of economic development, governance and publicly and privately funded health services. Recent evidence from China^11^ shows that a national policy increasing the proportion of health professionals per capita had a diverse impact on the reduction of child mortality in counties with different patterns of economic development.

Home to 210 million people and the world’s ninth largest economy, Brazil’s health system presents profound differences across its large territory and state division, which reflect not only their epidemiology and economic conditions but also the role played by the public and private sector in the provision of services.^12^ Brazil’s 27 federative units (26 states and the Federal Capital District) display very different conditions, with profound socioeconomic and health inequalities, which is typical of large and populous middle-income countries that include a population with diverse socio-historical backgrounds. Provision of health services in Brazil is organised through a combination of public funds, shared governance in the three federative levels, and diverse participation and relevance of private providers.^13 14^

Two Brazilian states neatly exemplify such differences. *São Paulo* state (SP) has one of the country’s highest per capita incomes—US$ 46 776 per month (or US$ 844 in purchasing power parity (PPP)), according to Brazilian Institute of Statistics and Geography (IBGE)—and 38% of its population is covered by private health schemes. The state concentrates the second largest group of individuals that have private health plans in the country (36%). The public expenditure on health is also among the highest, US$ 36 028 per capita in 2018 (US$ 650 PPP), considering states and municipalities spending based on their own sources and federal transfers. By contrast, per capita income in *Maranhão* state (MA) is one third of São Paulo’s, and the region concentrates a portion of only 1% of the national population covered by private health schemes (corresponding to 7% of the state’s population). Public health expenditures amount to US$ 22 541 per capita (US$ 407 PPP), among the lowest in the country.

Despite the constitutional provision for a publicly financed universal health system, private health plans play a significant role in the country: they receive substantial fiscal incentives and mostly cover a very heterogeneous group of mostly middle income customers that constitutes a quarter of the population.^15^ Health insurance coverage is critically linked to formal employment, as according to the National Agency of Supplementary Health (ANS), more than 2/3 of active contracts are part of remuneration packages. Historically, these plans are paid as a benefit in large companies and/or middle-class occupations. Thus, the coverage rate reflects the structure of the labour market, rather than variables related to individual purchasing power and spontaneous demand. Since 2000, the Constitutional Amendment n. 29 (*Emenda Constitucional,* EC29, 2000) led to a steady increase in the public sector’s share of total health spending, although the country’s Unified Health System (*Sistema Único de Saúde, SUS*) never exceeded 47% of total expenditure.^16^

Since 2011 Brazil experienced a profound social, political and economic crisis^17^ that turned into an economic recession by the end of 2014. The political scene also includes huge demonstrations since 2013, a controversial impeachment in 2016 and two hard-fought federal elections (2014 and 2018). Thus, multiple factors contributed to generate the most severe recession in Brazilian republican history.^18^ Recent literature states that the recent economic recession exacerbated the country’s pre-existing health inequalities,^19^ putting at risk the recent gains of its health system.^20^ The recently installed right wing governments are moving the country towards a more free-market economy; since 2016, economic measures have been introduced to reduce public spending—Constitutional Amendment n. 95 (EC95/2016)—and increase labour market flexibility—Labour Reform (Law n. 13.467/2017). Recent evidence has shown that the latest recession has contributed to an increase of mortality rates in the country.^21^

This paper is part of a wider study on the impact of Brazil’s current economic crisis on its health system and workforce.^22^ We argue that in many middle-income countries like Brazil, the impacts of economic recessions are felt unevenly across the health systems, depending not only on local economic conditions and the health system's pre-existing characteristics but also on the different policies adopted by local governments to offset the crisis’ effects. We set out to compare the macroeconomic and health financing, and system performance indicators before and after the 2015 economic recession of one of Brazil’s richest and most populated states (SP) with those of one of the country’s poorest (MA). It is hoped that the results from this comparison will help shed light on the different impacts that economic recessions can inflict on different parts of health systems in middle-income countries throughout the world and the consequent responses.

## Methods

**Figure 1:**
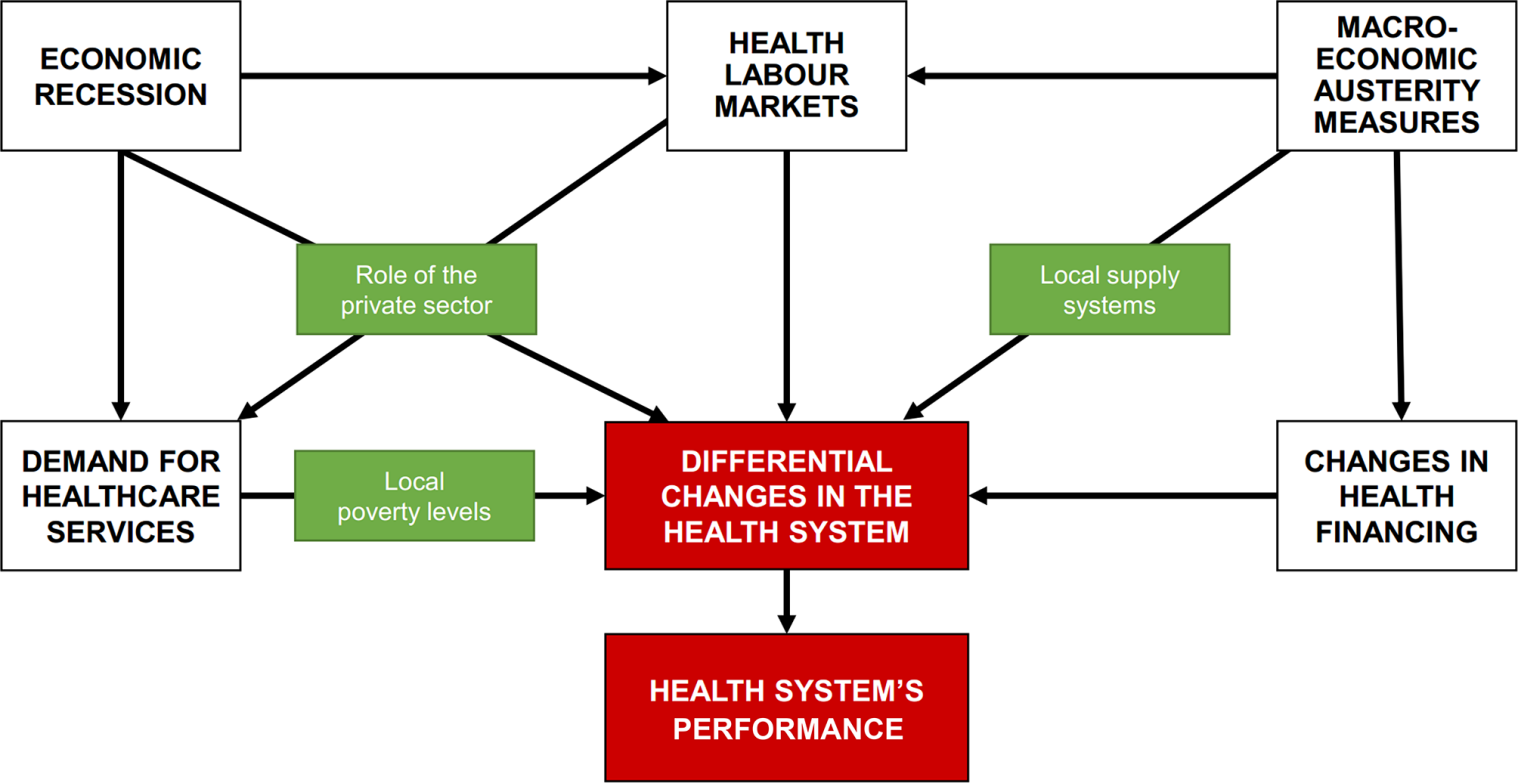
Conceptual framework.

Existing frameworks suggest that the impact of economic recessions are felt on population health and health systems through the combined increase of poverty-related diseases, deteriorating income and ability to pay, in addition to governments’ policy response to crises.^23–26^ Drawing from the literature of health system resilience in the face of economic shocks,^2 27 28^ we posit that a number of local factors and circumstances mediate the effects of recessions and austerity measures on health systems, at times mitigating the impact, in other cases exacerbating the overall consequences. We consider that local poverty and the stage of development of local health systems,^11^ the role played by the private sector^29^ and the structure of public financing for the health sector^2^ are resilience factors that can mitigate or exacerbate the effects of a crisis on systems and populations (figure 1).

By ‘differential changes in the health systems’ we imply that different parts of a national health system react differently to supposedly similar macro-effects of a recession, depending on local markets, local financing mechanisms or specific mitigating factors. This is why it is not possible to talk about a single effect from the crisis on a health system as diverse as Brazil’s, particularly when comparing states with substantially different underlying economic and development conditions like São Paulo and Maranhão.

Our literature-led hypothesis is that the impact from economic crises will be felt unevenly across the health system, as the pre-existing conditions and the role played by the private sector will mediate the effects of the crises. Differences in local financing mechanisms will also compensate the overall contraction of funds available for the health sector. The implication of these mediating factors will be visible in the uneven distribution of resources, and may show a distinctive regression in some contexts, but also resilience in others.

Following our conceptual framework and hypotheses earlier, we set out to explore the differential impact of Brazil’s economic slowdown in the two very different states of São Paulo and Maranhão. We use a comparative case-study approach^30^ to contrast the evidence available on the expected effects of the 2015–2016 economic contraction, on the supposed mediating factors and on the distribution of financial and non-financial resources across health systems in the two states.

As a large proportion of population and specialised healthcare institutions are typically hosted in Brazil’s state capitals, this paper also broke down the available health expenditure data by the two states’ capital cities—São Paulo city and São Luís de Maranhão. In addition to giving a better illustration of capital-country inequalities, this choice enriches the examination of low-level phenomena. Whenever possible, we also collected aggregate data on the municipality level, in particular budget indicators.

### Data and sources of information

In order to understand the relationship between the economic recession and the Brazilian health system, we began by selecting certain indicators to evidence both SP and MA socioeconomic positions. Gross domestic product (GDP) and per capita GDP indicators were extracted from the System of National Accounts.

Population, labour market and poverty indicators were taken from the National Household Sample Survey of the Brazilian Institute of Geography and Statistics. Health plans and insurance data—quantity of contracts, companies' income, among others—are published by the ANS. In this paper, ‘private health coverage’ signifies the number of existing health plan contracts divided by the country’s overall population.

Analysis regarding São Paulo and Maranhão was based on health expenditures recorded for each state and local governments before and after the crisis. In order to understand the general context, overall public spending was also examined, based on federal, state and local governments’ aggregate data extracted from the Public Health Budget Information System and complemented by fiscal statements.

Private health systems’ reaction to the crisis was described by analysing health plans and out-of-pocket expenditures. Company and family spending on health plans and the evolution of the number of beneficiaries were obtained from the ANS database. The analysis of household out-of-pocket expenditures, including drugs, services and other health items, was based on Health Satellite Account data collected by IBGE for the 2010–2015 period and estimated for the 2016–2018 period.

Health workforce data were extracted from the *Annual Relation of Social Indicators*, which exclusively covers formally employed physicians and nurses in the public health system (SUS). To describe the effects of the economic crisis on non-financial resources, we selected the evolution of intensive and intermediate hospital beds stock from the National Health Facilities Register; as the stock of such core beds is typically more stable than overall hospital beds, such an indicator is believed to better capture changes in this resource availability. These non-financial resources were weighted by the state population as estimated by IBGE.

### Data analysis

Historical expenditure data were analysed descriptively. Data originally collected in the current national currency (Brazilian Real) were deflated to average 2018 prices by Brazilian consumer price index and the whole series was then converted to US dollars at 2018 average exchange rate (US$ 1=R$ 3.66). This methodological approach was intended to correct national health spending for the distortions introduced by the substantial exchange rate fluctuations experienced by Brazil during the period under analysis.

In order to allow a comparative glance, some values in our analysis were also expressed in parenthesis using the PPP approach, available from the World Bank Open Data. Such methodology had been used before in studies focusing on comparative health spending across countries.^31^ The exchange rate for 2018 is US$ 1=R$ 2.03.

### Patient and public involvement

Patients or the public were not involved in the design, conduct, reporting or dissemination plans of our research.

## Results

### The socio-economic positions before and after the crisis

This section sets forth a comparison between Brazil, MA and SP (and their respective capital cities, *São Luís* and *São Paulo*), and the effects of the crisis on selected indicators. It presents information on changes in macroeconomic indicators before and after the crisis in the following settings for three key dimensions: GDP and poverty, the (health) labour market, and health insurance coverage for the 2010–2018 period.

#### Decreasing GDP and increasing poverty

Currently São Paulo has the largest GDP among all 27 Brazilian states—almost 1/3 of the total Brazilian GDP. Maranhão ranks 17th, with 1.4% of the total GDP. It is important to note the capitals’ GDP share (around 30% in both states). The city of São Paulo accounts for 11% of the national GDP.^32^ Disparities between MA and SP are also reflected by per capita GDP (table 1). Among all states in Brazil, MA has the lowest rate and SP holds the second position. Again, both capitals stand out: the per capita GDP of São Luís is 2.5 times larger than the state average, which highlights the different distribution of income in the population in the two states. The latest poverty data show that more than half of MA’s population lives below the poverty line, which in Brazil is half the national minimum salary. In SP, this poverty indicator varies between 10% and 20% in the selected period.

**Table 1:** Selected socioeconomic indicators (2010–2018)

GDP	2010	2011	2012	2013	2014	2015	2016	2017	2018
US$ billions									
Brazil	1696	1804	1863	1950	1995	1946	1837	1809	1865
MA	20	22	23	25	27	25	25	—	—
São Luís (MA)	8	8	9	8	10	9	8	—	—
SP	565	592	603	627	642	630	598	—	—
São Paulo (SP)	197	207	208	213	215	212	201	—	—

GDP per capita	2010	2011	2012	2013	2014	2015	2016	2017	2018
US$ billions*									
Brazil	8860	9377	9611	9698	9841	9516	8916	8714	8947
MA	3175	3234	3487	3643	3873	3688	3596	—	—
São Luís (MA)	7973	8005	8443	8027	8860	8097	7668	—	—
SP	13 658	14 240	14 401	14 365	14 570	14 178	13 353	—	—
São Paulo (SP)	17 818	18 285	18 333	18 005	18 051	17 722	16 733	—	—

Absolute poverty†	2010	2011	2012	2013	2014	2015	2016	2017	2018
Percentage									
Brazil	—	28.2	28.3	27.6	25.4	27.4	29.9	30.7	—
MA	—	58.2	58.8	53.5	53.3	54.1	58.0	59.5	—
São Luís (MA)	—	—	—	—	—	—	12.5	—	—
SP	—	13.6	13.6	13.7	11.9	14.3	15.4	18.3	—
São Paulo (SP)	—	—	—	—	—	—	3.7	—	—

Unemployment rate	2010	2011	2012	2013	2014	2015	2016	2017	2018
Percentage									
Brazil	—	—	6.9	6.2	6.5	9.0	12.0	11.8	11.6
MA	—	—	7.5	5.5	7.0	8.2	13.0	13.3	14.1
São Luís (MA)	—	—	12.3	6.7	11.3	13.6	17.6	19.8	15.9
SP	—	—	6.8	6.5	7.1	10.1	12.4	12.7	12.4
São Paulo (SP)	—	—	6.2	6.0	6.4	8.9	12.3	13.4	13.6

Subemployment rate‡	2010	2011	2012	2013	2014	2015	2016	2017	2018
Percentage									
Brazil	—	—	16.7	14.9	14.9	17.3	22.2	23.6	23.9
MA	—	—	21.8	20.1	21.9	23.2	34.2	35.8	38.4
São Luís (MA)	—	—	17.2	10.5	15.4	19.7	21.7	27.3	23.0
SP	—	—	11.7	10.8	11.8	15	18.9	20.4	20.5
São Paulo (SP)	—	—	9.5	10.7	10.1	13.9	18.7	20.2	21.1

Private health coverage	2010	2011	2012	2013	2014	2015	2016	2017	2018
Percentage									
Brazil	22.3	23.4	23.8	24.6	25.5	25.9	25.3	24.4	24.2
MA	4.6	5.3	5.8	6.3	7.1	7.2	7.2	6.8	6.8
São Luís (MA)	20.0	23.0	25.3	28.2	31.4	31.2	30.7	29.1	28.6
SP	40.7	41.8	42.0	43.0	44.2	44.5	43.4	41.3	40.9
São Paulo (SP)	53.9	54.4	55.4	56.3	57.3	57.4	56.0	52.3	50.7

Source: Brazilian Institute of Statistics and Geography—Sistema de Contas Nacionais. Estimativas de População. Pnad Contínua. Síntese de Indicadores Sociais. Brazilian Consumer Price Index; National Agency of Supplementary Health; Public Health Budgets Information System.

*Constant values in BRL converted to 2018 USD.

†Ratio of people living in households where the per capita income is lower than 1/2 Brazilian minimum wage.

‡The subemployment rate includes the unemployed, demotivated workers that are no longer seeking work, and part-timers who would like to work more hours.

The indicators presented in table 1 show a downward turn between 2014 and 2015. GDP slowed down since the beginning of the period, and increased until 2014 before explicitly becoming a recession. This trend affects both states and cities. The effects of the recession are noticeable in the changes in GDP per capita, which peaks in 2014 nationwide, then contracts by 10% around 2018. Absolute poverty increases by 20% between 2014 and 2017, and is more prevalent in SP than in MA in relative terms (50% vs 12%, respectively), although from very different starting points.

#### The national labour market during the crisis: evolution of unemployment and subemployment

The Brazilian labour market is characterised by high rates of informal unemployment and labour turnover, which is related to the substantial presence of flexible, insecure, and precarious jobs.^33^ Table 1 shows the unemployment rate for each location; in comparison, the change in the proportion of active population in employment displays a similar behaviour for all locations but São Luís, where this indicator is more erratic (lowest 6.7% in 2013, highest 18.8% in 2016). The subemployment rate (which includes demotivated and involuntary part-time workers) is markedly different for MA and SP, and between the respective capitals; in MA, the gap between unemployment and subemployment exceeded 14% in 2012, whereas in SP it was 4.9%, which is indicative of fewer regular long-term jobs in MA.

Unemployment starts to grow in 2014 in all settings and reaches 14.1% in MA in 2018 and 12.4% in SP, with particularly high levels in urban areas. This effect is particularly evident in Maranhão’s capital city, São Luís. Since 2017, unemployment retreats slightly and unevenly among the locations, which led many to believe that the crisis was close to its end. The subemployment rate paints a more nuanced context. Between 2015 and 2018 this indicator has increased substantially for the whole country, reaching 24% (about 27 million people), which is the highest indicator value to date. Both MA and SP follow the national trend, including their capitals. Maranhão stands out for its high subemployment rate among all the states in Brazil, 38.4%, which is particularly high in the state's countryside. Unlike unemployment, which seemed to stabilise after 2017, subemployment continues to grow, and raises doubts about optimistic analyses concerning labour market recovery. Furthermore, data suggest unemployed workers are getting discouraged or accepting jobs offering conditions worse than before the recession. In addition, most recent data indicate lasting stagnation for 2019.

#### Decline of private health insurance coverage

In Brazil, health insurance coverage is critically linked to formal employment. Our macroeconomic data (table 1) emphasise this characteristic and highlights important shifts of the last decade, as the country’s coverage rate is currently around 25%, growing until 2014. As a lagged effect of unemployment, private health insurance coverage starts contracting in 2016 (−6.5% nationally in the next 2 years), with particularly noticeable effects in SP (−10%) and its capital city, but not as noticeable in MA state. SP had an intense growth between 2010 and 2014, overcoming the national average. MA has a very low coverage rate (about 7%) which reflects the region’s socioeconomic condition and its labour market structure. As expected, both capitals have higher coverage rates than the state level, but also show a slower dynamic before 2015, suggesting that in the last years, health insurance companies have expanded in smaller cities in the countryside. The city of São Paulo alone has more than half of all active health plans in Brazil. This reflects higher participation of major companies and upper-level civil servants, which are groups traditionally covered by this modality of supplementary healthcare.^34^

The private coverage indicator also matches the proposed periodisation. A momentary decrease in the number of contracts is an expected result of the economic crisis, notwithstanding the upward trend of this market. As demonstrated by the data series, there is a common turning point in Brazil for both states and its capitals between 2014 and 2015 and and after 2016, the fall is even more pronounced.

### Health financing in Brazil, Maranhão and São Paulo before and after the crisis

Considering the available data for public and private health spending, overall health spending steadily increased in Brazil in the 2010–2018 period except for 2016; however, in 2017, per capita expenditures had still not reached 2014 levels. Having increased regularly over the pre-crisis years, the public sector generally kept its real terms value, slumping in 2015 and 2016, but recovered its pre-crisis spending levels in 2017–2018. Private spending, which before the economic recession presented substantial real increases, exhibits an uneven pattern in the subsequent period, since out-of-pocket expenditures reduced after the crisis, whereas health plans increased by 22% between 2015 and 2018 (table 2).

**Table 2:** Evolution of private and public health spending (2010–2018)

Health spending by source (US$ billions*)		2010	2011	2012	2013	2014	2015	2016	2017	2018	Average change 2010–2014	Average change 2015–2018
**Public**	**Total public spending**	**61.7**	**66.8**	**70.1**	**72.9**	**76.2**	**75.2**	**73.5**	**75.9**	**75.5**	**5.4%**	(**0.2%**)
	**Growth rate**		**8.3%**	**4.9%**	**4.0%**	**4.5%**	(**1.3%**)	(**2.2%**)	**3.2%**	(**0.5%**)		
	**% of total spending**	**45.8**	**46.7**	**45.8**	**45.1**	**44.0**	**43.4**	**43.4**	**43.6**	—		
	Federal (direct+transfers)	**27.4**	**29.9**	**31.4**	**30.7**	**32.0**	**31.9**	**31.2**	**32.5**	**32.0**	**4.0%**	**0.0%**
	States (own sources)	**17.0**	**17.9**	**18.3**	**20.1**	**20.8**	**20.2**	**19.4**	**20.2**	**20.8**	**5.2%**	**0.0%**
	Local (own sources)	**17.3**	**19.0**	**20.4**	**22.2**	**23.4**	**23.0**	**23.0**	**23.2**	**22.7**	**7.8%**	(**0.8%**)
**Private**	**Total private spending**	**73.0**	**76.4**	**83.0**	**88.7**	**97.0**	**97.9**	**95.8**	**98.0**	—	**7.4%**	—
	**Growth rate**	–	**4.6%**	**8.6%**	**7.0%**	**9.3%**	**1.0%**	(**2.2%**)	**2.4%**	—		
	**% of total spending**	**54.2**	**53.3**	**54.2**	**54.9**	**56.0**	**56.6**	**56.6**	**56.4**	—		
	Health plans	**32.8**	**34.9**	**37.4**	**40.3**	**44.0**	**45.7**	**47.4**	**50.8**	**53.5**	**7.6%**	**5.0%**
	Out of pocket	**40.2**	**41.5**	**45.6**	**48.4**	**53.0**	**52.2**	**48.4**	**47.2**	—	**7.1%**	—
**Total spending**		**134.7**	**143.2**	**153.1**	**161.7**	**173.2**	**173.1**	**169.3**	**173.9**	—	**6.5%**	—
**Growth rate**			**6.3%**	**6.9%**	**5.6%**	**7.1%**	**0.0%**	(**2.2%**)	**2.7%**	—		
**Total spending per capita**		**703.6**	**744.6**	**789.6**	**804.2**	**854.0**	**846.8**	**821.6**	**837.5**	—	**5.0%**	—
**Public spending per capita**		**322.2**	**347.5**	**361.7**	**362.8**	**375.7**	**367.8**	**356.9**	**365.4**	**362.2**	**3.9%**	(**0.9%**)
**Health plans spending per capita**		**729.4**	**758.8**	**781.5**	**814.7**	**872.5**	**929.0**	**995.5**	**1078.9**	**1132.5**	**4.6%**	**6.7%**

Source: Public data—Ministry of Health/Public Health Budgets Information System; private data—Brazilian Institute of Statistics and Geography, National Agency of Supplementary Health.

*Constant values in BRL converted to 2018 USD.

Government expenditure at all federative levels increased tremendously in real terms during the economic growth period (2010–2014), particularly at the local governments level. Between 2010 and 2014, municipality spending reached growth rates even higher than private spending items, whereas from 2015 onwards, expenditure registered real loses in the context of tax revenue weakness. For private sources, expenditures on health plans had solid growth rates after the crisis, despite having lost more than three million beneficiaries since 2014. As a result, per capita expenditures on private health plans, which before the economic crisis were roughly twice as much as public expenditure, reached more than three times that value in 2018.

Since the economic recession, total federal government health spending fluctuated around 2014 levels, after the steady increase in real terms experienced the previous decade. After the crisis, direct federal expenditure maintained a trend of expansion, although at a much slower rate than in the previous period (table 3). Transfers to local governments, which grew between 2010 and 2014, were reduced until 2016, and then slightly recovered. Contractions in state transfers characterised the whole period. The proportion of the budget absorbed by capital cities does not appear to change dramatically, as historical funding allocation is ‘political’ in Brazil, and the austerity cuts have mostly been proportional for states and capital cities.

**Table 3:** Public only health spending and transfers by federative level: federal, state and local (2010–2018)

Health spending by source (US$ billions*)		2010	2011	2012	2013	2014	2015	2016	2017	2018	Average change	
											2010–2014	2015–2018
**Federal government**	**Total**	**27.35**	**29.94**	**31.45**	**30.71**	**31.96**	**31.91**	**31.16**	**32.52**	**31.95**	**4.0%**	**0.0%**
	**Direct**	**7.62**	**8.75**	**9.45**	**9.76**	**10.55**	**11.27**	**11.45**	**11.47**	**11.04**	**8.5%**	**1.1%**
	**Transfers**	**19.73**	**21.19**	**22.00**	**20.95**	**21.41**	**20.64**	**19.71**	**21.05**	**20.91**	**2.1%**	(**0.6%**)
	States	6.82	6.45	6.87	6.38	5.92	5.49	5.25	5.49	5.27	(3.5%)	(2.9%)
	Local governments	12.91	14.75	15.13	14.57	15.49	15.15	14.46	15.56	15.64	4.7%	0.2%
**São Paulo**	**Total (state+local**)	**14.32**	**14.95**	**15.38**	**16.22**	**16.62**	**16.26**	**16.08**	**16.47**	**16.44**	**3.8%**	(**0.3%**)
	**State total**	**6.30**	**6.35**	**6.11**	**6.37**	**6.20**	**6.03**	**5.74**	**6.14**	**6.12**	(**0.4%**)	(**0.3%**)
	Own sources	4.43	4.66	4.61	4.84	4.67	4.48	4.36	4.68	4.60	1.3%	(0.4%)
	Transfers	1.87	1.70	1.49	1.53	1.53	1.56	1.39	1.46	1.53	(4.9%)	(0.1%)
	**Local governments total**	**8.02**	**8.59**	**9.27**	**9.86**	**10.43**	**10.22**	**10.34**	**10.33**	**10.32**	**6.8%**	(**0.3%**)
	Own sources	5.84	6.33	6.76	7.30	7.57	7.53	7.59	7.70	7.46	6.7%	(0.4%)
	Transfers	2.18	2.26	2.51	2.56	2.85	2.69	2.75	2.63	2.85	7.0%	0.0%
**São Paulo (capital**)	**São Paulo (capital) Total**	**2.24**	**2.35**	**2.47**	**2.52**	**2.64**	**2.73**	**2.85**	**2.85**	**2.73**	**4.2%**	**0.8%**
	Own sources	1.69	1.80	1.90	1.88	1.96	2.05	2.21	2.26	2.13	3.7%	2.0%
	Transfers	0.55	0.55	0.58	0.64	0.68	0.67	0.63	0.59	0.61	5.5%	(2.9%)
**Maranhão**	**Total (state+local**)	**1.30**	**1.41**	**1.44**	**1.54**	**1.62**	**1.49**	**1.49**	**1.60**	**1.71**	**5.6%**	**1.3%**
	**State total**	**0.41**	**0.47**	**0.53**	**0.53**	**0.58**	**0.55**	**0.54**	**0.58**	**0.61**	**9.0%**	**1.3%**
	Own sources	0.32	0.37	0.39	0.40	0.45	0.43	0.42	0.46	0.48	8.5%	1.6%
	Transfers	0.08	0.11	0.14	0.13	0.13	0.11	0.12	0.12	0.13	10.8%	0.2%
	**Local governments total**	**0.90**	**0.94**	**0.91**	**1.00**	**1.05**	**0.94**	**0.95**	**1.02**	**1.10**	**4.0%**	**1.3%**
	Own sources	0.36	0.39	0.36	0.43	0.44	0.41	0.42	0.45	0.43	5.3%	(0.9%)
	Transfers	0.53	0.55	0.55	0.58	0.60	0.53	0.53	0.57	0.68	3.1%	2.8%
**São Luís (capital**)	**São Luis total**	**0.21**	**0.23**	**0.21**	**0.24**	**0.24**	**0.21**	**0.24**	**0.24**	**0.24**	**3.4%**	**0.7%**
	Own sources	0.11	0.12	0.11	0.14	0.13	0.12	0.14	0.13	0.13	6.0%	(0.9%)
	Transfers	0.10	0.11	0.10	0.11	0.10	0.09	0.09	0.10	0.11	0.3%	2.6%

Source: Public data—Ministry of Health/Public Health Budgets Information System; private data—National Agency of Supplementary Health.

*Constant values in BRL converted to 2018 USD.

When analysing health spending for all of Brazil, it is noticeable how public expenditure did not grow in the 4 years (2015–2018) after the economic crisis in Brazil, whereas it had grown by an average rate of 5% per year in real terms during the 4 years before the crisis. The same trend is reflected for São Paulo state, where public health spending grows by an average of 1% in the last 4 years, with a substantial slump around 2015 and 2016. In São Paulo city, the capital’s own resources propped up growth both during and after the crisis.

Health spending in Maranhão is roughly 1/10th of São Paulo’s and the state government increased spending substantially (average growth of 9% in real terms) in the 4 years before the crisis and experienced a substantial slump in funding for 2015 and 2016. Local government’s funding followed state expenditure, and showed a slightly more pronounced increase in federal transfers in recent years (table 3). Maranhão’s capital city, São Luís, benefitted particularly from this trend, as its public spending grew at a faster pace in the 4 years after the crisis (2.6%) than before (0.3%).

### Changes in non-financial resources available to the health systems

The data on health personnel per population in the public sector (SUS) show an overall decrease of physicians (−10%) across the country from 2012 to 2017; however there is an overall increase of nurses (26%). Physician levels peak between 2014 and 2015 in all settings, before decreasing marginally. This trend for physicians in the public sector was particularly acute in São Paulo state, as a 25% decrease was observed in the availability of doctors per capita, while in Maranhão the proportion remained stable. Conversely, the nursing workforce per capita increased across the board despite the economic recession, particularly in Maranhão, which had a 53% increase across the 6 years. No specific pattern of fluctuation was visible for the years before and after the economic crisis.

As per intensive care beds per capita in the public sector, no significant change is visible between 2012 and 2017 in the three settings, as pre-crisis levels of beds to population ratios were maintained (table 4). The stability of intensive beds contradicts the general decreasing trend of overall (non-intensive) beds in public and private sector; this may be explained by the evolution of the hospitalisation and treatment model in Brazil over the last 10 years where hospital capacity has been reduced to accompany a shift to outpatient care.^35^

**Table 4:** Public health personnel and hospital beds per capita (2012–2017)

Resources *Density per 1000 people*	Location	2012	2013	2014	2015	2016	2017
Physicians formally employed in SUS	**Brazil**	**1.43**	**1.34**	**1.33**	**1.33**	**1.28**	**1.29**
	Maranhão	0.27	0.32	0.30	0.27	0.25	0.28
	São Paulo	2.18	1.77	1.81	1.75	1.71	1.64
Nurses formally employed in SUS	**Brazil**	**1.13**	**1.20**	**1.30**	**1.34**	**1.35**	**1.42**
	Maranhão	0.49	0.57	0.63	0.51	0.61	0.75
	São Paulo	1.51	1.59	1.72	1.77	1.80	1.85
Intensive+intermediate SUS beds	**Brazil**	**0.14**	**0.13**	**0.14**	**0.14**	**0.14**	**0.14**
	Maranhão	0.09	0.10	0.10	0.11	0.11	0.11
	São Paulo	0.16	0.15	0.15	0.15	0.15	0.16

Source: Annual Relation of Social Indicators, National Health Facilities Register.

## Discussion

Our analysis shows that Brazil’s macroeconomic conditions deteriorated considerably in 2015–2016, as GDP per capita decreased substantially in Maranhão and São Paulo: it is unclear whether the economy in the two states has returned to pre-crisis levels. Initially considerably richer, São Paulo’s economy seems to have experienced a relatively larger setback than Maranhão’s, despite Maranhão’s poverty levels continuing to be drastically above the national average. Unemployment and subemployment increased significantly in the two states, and the capital cities felt the largest effects; changes in employment levels affected the private health insurance market, as its population coverage decreased accordingly. In connection with the economic crisis, public health expenditures flattened in Brazil after 2015, while private health spending related to health plans significantly increased.

Health financing patterns differed in the two states and capital cities, as public health expenditures in Maranhão continued to grow after the crisis years, although at a slower rate, propped up by the increase in federal transfers to local government funding. Contrarily from richer São Paulo, this substitution of local for central public expenditures in Maranhão allowed health spending to bounce back to growth rates experienced before the crisis. As a consequence of the macroeconomic, public and private spending changes seen in the two states, public sector staffing levels and beds per capita did not appear to have been exceedingly affected by the crisis in Maranhão, whereas a decrease of public sector physicians was indeed observed in São Paulo after 2015.

The increase of private health spending during and after the economic recession is a somewhat surprising finding from our analysis, particularly as many people lost their plans in connection to the rising unemployment. A possible explanation may be that, precisely because of the uncertainty brought on by the recession, those who could, decided to over-insure against the increased risk, which is something that was observed in Portugal too during the Great Recession.^36^ Or perhaps, as reforming public health spending during the crisis opened up new opportunities for the private sector, private providers found themselves in a position to induce demand to compensate for the loss of customers.^37^

Contrary to expectations, we found that overall public health spending in Brazil did not contract irreversibly during the economic recession, as it rebounded in the immediate aftermath for the states in aggregate. The effects of such a trend were visible in Maranhão considering the non-financial health resources—human resources and beds per capita—that largely remained steady in the public healthcare system for the last ten years. This care capacity resistance was true even for São Paulo, a state whose expenditures in 2018 were still below 2014 rank in real terms. In Maranhão, state funds and federal transfers maintained positive real increases after the crisis, whereas local governments own sources registered negative average variation during the 2014–2018 period. Such findings seem to be consistent with studies showing that fiscal decentralisation can lead to increases of public health spending even during economic and financial crises,^38^ and, if confirmed, would lend support to those authors that have advocated the benefits of fiscal federalism for social sectors for other countries.^39^

When contrasting the situation in São Paulo and Maranhão, our data suggest the crisis has had a diverse impact in both states. Starting from a much higher income level, in São Paulo the macroeconomic slowdown has been more intense, with greater job losses, and setting back the state to the last decade’s levels of poverty. Health expenditures decreased, and 2018 figures were below 2014 levels in real terms. Linked to unemployment, private insurance coverage slipped, and it has not recovered its pre-crisis levels. Private spending data did not allow analysis at a sub-national level, but, given the trend, it does not seem outlandish to imagine that private health spending will have increased in São Paulo during the crisis because of the expenditures in health insurance. In Maranhão, however, the crisis seems to have been felt less intensely; starting from a lower base, the macroeconomic slowdown was less noticeable, although it did increase poverty levels. Public health spending maintained its level during the crisis, sustained by the state’s own resources and decentralised funds, and private insurance coverage kept steady.

Despite the peculiarities of Brazil’s health system and the unique features of its economic crises, our analysis bears implications for health financing and provision of health services amid economic slowdowns in low/middle-income settings. This ‘differential impact’ of crises on health sectors is not new, as the concept of a system’s vulnerability stemming from the combination of its exposure to economic fluctuation and its ability to respond to change is well-established in the development economic literature.^40^ Paradoxically, our results show that more dynamic and sophisticated health systems, reliant on multiple health services providers, may find themselves more exposed during an economic downturn than poorer systems that rely predominantly on less volatile public funds. In such a scenario, public health spending would create mechanisms of social protection during an economic downturn, therefore counteracting the expected disproportionate impact of recessions on services for the poor.^41^ This conclusion appears consistent with the recent evidence from Brazil on the relation between unemployment and health during recession, and on the mitigation effects of social protection mechanisms.^42^

Our findings call for a more nuanced understanding of how the effects of economic recessions reverberate across complex and multi-layered health systems typical of the world’s emerging middle-income economies. Our results should be interpreted in the light of the study’s acknowledged limitations. First, public health expenditures in Brazil take place within a complex federative structure, including financial transfers between central, state and local governments. Therefore, tracking expenditures is complex, and the risk of double counting expenditures is very real, as these are often recorded by multiple agencies at different levels of government. This possible bias should not however change the discussed expenditure trends. Second, the lack of information on the private sector hampered our exploration of its behaviour during the economic recession; more complete data on out-of-pocket spending for medicines, medical services and other markets would strengthen our analysis and conclusions. Third, our methodological choice to convert national expenditure in US dollars may have, to some extent, distorted the spending trends, as during the period of study, BRL to USD exchange rate had non-negligible fluctuations, which may have influenced some of our conclusions from a cross-countries comparative perspective. Finally, the analysis must take into account that both MA and SP are large territories with great heterogeneities between and within cities, which may not have been fully captured by our aggregated data. Despite such limitations, a few key lessons can be safely learnt from our study.

## Conclusion

Economic recessions are common phenomena in high/low-income settings, but their impact on world health systems is still unclear. We designed a conceptual framework to analyse the macroeconomic effects of recessions to labour markets, the demand and supply of health services and health system performance in two Brazil’s states.

Our analysis shows that Brazil’s broader macroeconomic conditions deteriorated substantially after 2015–2016, and São Paulo’s economy experienced a larger setback than Maranhão’s. In connection with the economic crisis, we showed how public health expenditures flattened in Brazil, while private health insurance spending significantly increased, whereas out-of-pocket expenditures reduced in real terms. Public health financing patterns also differed across the two states, as public health funding in Maranhão continued to grow after the crisis years, propped up by the increase in transfers to local government funding. Despite the recession, staffing levels and beds per capita in the public sector were not exceedingly marked by the crisis in Maranhão, whereas a decrease in the number of physicians was indeed observed in São Paulo.

Our case study suggests that the effects of a recession in LMICs may not be immediately felt across a health system, as they are mediated by private spending and by adjustments in public financing, and according to the role played by the private sector in the provision of services, and the local social protection policies and health financing arrangements. We call for a more refined understanding of the differential impact of economic recessions on health systems, with the objective of identifying effective policies to mitigate their effects.
